# Immunoinformatic-Based Multi-Epitope Vaccine Design for Co-Infection of *Mycobacterium tuberculosis* and SARS-CoV-2

**DOI:** 10.3390/jpm13010116

**Published:** 2023-01-05

**Authors:** Cong Peng, Fengjie Tang, Jie Wang, Peng Cheng, Liang Wang, Wenping Gong

**Affiliations:** 1 Tuberculosis Prevention and Control Key Laboratory/Beijing Key Laboratory of New Techniques of Tuberculosis Diagnosis and Treatment, Senior Department of Tuberculosis, Eighth Medical Center of PLA General Hospital, 17^#^ Heishanhu Road, Haidian District, Beijing 100091, China; 2 Department of Geriatrics, Eighth Medical Center of PLA General Hospital, 17^#^ Heishanhu Road, Haidian District, Beijing 100091, China; 3 Graduate School, Hebei North University, Zhangjiakou 075000, China; 4 Department of Respiratory Medicine, Chongqing Emergency Medical Center, Chongqing University Central Hospital, Chongqing 400010, China

**Keywords:** *Mycobacterium tuberculosis* (MTB), SARS-CoV-2, multi-epitope vaccine (MEV), bioinformatics, immunoinformatics

## Abstract

(1) Background: Many co-infections of *Mycobacterium tuberculosis* (MTB) and severe acute respiratory syndrome coronavirus-2 (SARS-CoV-2) have emerged since the occurrence of the SARS-CoV-2 pandemic. This study aims to design an effective preventive multi-epitope vaccine against the co-infection of MTB and SARS-CoV-2. (2) Methods: The three selected proteins (spike protein, diacylglycerol acyltransferase, and low molecular weight T-cell antigen TB8.4) were predicted using bioinformatics, and 16 epitopes with the highest ranks (10 helper T lymphocyte epitopes, 2 CD8^+^ T lymphocytes epitopes, and 4 B-cell epitopes) were selected and assembled into the candidate vaccine referred to as S7D5L4. The toxicity, sensitization, stability, solubility, antigenicity, and immunogenicity of the S7D5L4 vaccine were evaluated using bioinformatics tools. Subsequently, toll-like receptor 4 docking simulation and discontinuous B-cell epitope prediction were performed. Immune simulation and codon optimization were carried out using immunoinformatics and molecular biology tools. (3) Results: The S7D5L4 vaccine showed good physical properties, such as solubility, stability, non-sensitization, and non-toxicity. This vaccine had excellent antigenicity and immunogenicity and could successfully simulate immune responses in silico. Furthermore, the normal mode analysis of the S7D5L4 vaccine and toll-like receptor 4 docking simulation demonstrated that the vaccine had docking potential and a stable reaction. (4) Conclusions: The S7D5L4 vaccine designed to fight against the co-infection of MTB and SARS-CoV-2 may be safe and effective. The protective efficacy of this promising vaccine should be further verified using in vitro and in vivo experiments.

## 1. Introduction

Tuberculosis (TB) is an infectious disease mainly transmitted by the respiratory tract, and its pathogen is *Mycobacterium tuberculosis* (MTB) [1,2]. According to the Global Tuberculosis Report 2022 released by the World Health Organization, an estimated 10.6 million new cases of TB and 1.4 million TB-related deaths occurred among human immunodeficiency virus-negative people in 2021 [3]. In addition, the emergence of the coronavirus disease-2019 (COVID-19), caused by severe acute respiratory syndrome coronavirus-2 (SARS-CoV-2), may have negatively impacted TB prevention, diagnosis, and treatment [3]. Furthermore, a growing number of co-infections of MTB and SARS-CoV-2 have been reported in previous studies [4,5]. Mortality from co-infection with MTB and SARS-CoV-2 was reported to be markedly higher in older adults than younger patients; however, the reasons for this remain unclear [6]. Therefore, a novel vaccine against co-infection with MTB and SARS-CoV-2 is necessary [7].

Previous studies have shown that the antigen diacylglycerol acyltransferase (Ag85a, Rv3804c) is a candidate antigen for developing a vaccine against MTB infection [8]. A multicenter, double-blind, randomized, placebo-controlled phase II clinical trial has evaluated the safety and immunogenicity of the Ag85a antigen (NCT03878004), and the results showed that Ag85a had a higher median fusion response, and 94% of the volunteers responded to Ag85a [9]. Furthermore, the low molecular weight T-cell antigen, TB8.4 (Mtb8.4, Rv1174c) is another possible vaccine candidate. A previous study found that a multi-stage MTB subunit vaccine, LT70, consisting of the Mtb8.4 antigen induced a long-term protective effect against MTB [10]. In addition, the Mtb8.4 antigen showed good immunogenicity, induced robust cellular and humoral responses, and resulted in a protective effect against MTB infection [11,12,13].

The SARS-CoV-2 gene encodes both structural and non-structural proteins [14]. Four structural proteins are critical in the assembly and invasion of the SARS-CoV-2 spike protein, which is one of the structural proteins and constitutes spikes on the surface of the virus, which attach to host cells by binding to their receptors [14]. Meanwhile, spike protein can also be attached to target cells by binding to human angiotensin-converting enzyme 2 [15], suggesting that vaccines based on spike proteins can induce antibodies to block the binding and fusion of SARS-CoV-2 or neutralize viral infection [16,17,18]. Therefore, the Ag85a and Mtb8.4 antigens of MTB and the spike protein of SARS-CoV-2 were selected to construct a novel multi-epitope vaccine (MEV) in this study.

Traditional vaccine development methods are time-consuming and labor-intensive [19]. However, the emergence of bioinformatics and bioinformatics technology has changed the conventional vaccine construction strategy, making vaccine research and development more efficient and economical [20,21]. In recent years, an increasing number of MEVs have been successfully developed using bioinformatics and bioinformatics tools [22,23,24,25].

Therefore, this study employed bioinformatics and bioinformatics tools to predict the potential immunodominant epitopes of the MTB antigen and SARS-CoV-2 spike protein. Further, an MEV was designed, and its structural, immunological, and chemical characteristics were analyzed. As a result, this study provides a new vaccine candidate for the prevention of MTB and SARS-CoV-2 co-infection.

## 2. Materials and Methods

### 2.1. Sequence and Structure Retrieval

The sequences of the spike glycoprotein of SARS-CoV-2 (ID: P0DTC2), Ag85a of MTB (ID: P9WQP3), and Mtb8.4 of MTB (ID: O50430) in FASTA format were obtained from the UniProt database (https://www.uniprot.org/, accessed on 15 October 2022). These proteins were selected based on their excellent antigenic potential and essential role in immune activation against MTB infection [1,2].

### 2.2. Prediction of Linear B-Cell Epitopes

B cells and humoral immunity play an important role in host defense against MTB by affecting the development of immune responses [26]. The ABCpred server (https://webs.iiitd.edu.in/raghava/abcpred/ABC_submission.html, accessed on 15 October 2022) was used to predict linear B-cell epitopes from the selected antigens. This server effectively predicts linear B-cell epitope regions using artificial neural networks and has been widely applied in vaccine research [27]. The parameters were set as follows: epitope length = 16, and screening threshold = 0.51. The epitopes with scores greater than the threshold were selected and eventually used as dominant B-cell epitopes to construct candidate vaccines.

### 2.3. Prediction of Helper T-Lymphocyte (HTL) Epitopes

The Immune Epitope Database (IEDB) includes samples from non-human primates, humans, and other animal species. The major histocompatibility complex (MHC)-II server (http://tools.iedb.org/mhcii/, accessed on 16 October 2022) in the IEDB database was used in this study to predict HTL epitopes, as in our previous studies [1,24,25]. The parameters were set as follows: prediction method = IEDB recommendation 2.22, MHC allele = human leukocyte antigen (HLA) reference set (HLA-DP, HLA-DR, HLA-DQ), MHC source species = human, and epitope length = 15. Next, potential HTL epitopes were selected using a percentile rank score method. The lower the percentile rank score, the higher the epitope binding to MHC-II. Epitopes with percentile levels less than 0.5 were selected for further analysis. Then, the IFN-γ inducibility of selected epitopes was predicted using the IFN-γ Epitope Server (http://crdd.osdd.net/raghava/ifnepitope/index.php, accessed on 16 October 2022) [28]. According to the prediction results, the epitopes with positive prediction values were selected. Finally, VaxiJen v2.0 (http://www.ddg-pharmfac.net/vaxijen/VaxiJen/VaxiJen.html/, accessed on 16 October 2022) was employed to predict the antigenicity of selected epitopes with a threshold set at 0.5 [29]. Epitopes that passed the above screening were identified as immunodominant HTL epitopes for vaccine construction.

### 2.4. Prediction of Cytotoxic T-Lymphocyte (CTL) Epitopes

The detection of MHC-I antigenic peptides exposed to target cells and recognized by cytotoxic CTLs is essential for adaptive immune responses. Therefore, the IEDB MHC-I server was employed to predict CTL epitopes for the three associated antigens. This server (http://tools.iedb.org/mhci/, accessed on 18 October 2022) predicts the epitopes bound to MHC-I molecules and uses the artificial neural network 4.0 to represent 36 of HLA-A alleles, 34 of HLA-B alleles, and 10 of HLA-C alleles [30]. According to the percentile rating, the CTL epitopes with a threshold higher than 0.5 were selected for subsequent analysis. Then, the VaxiJen v2.0 server was employed to analyze the antigenicity of the above epitopes, and the threshold was set as 0.5, according to a previous study [29]. Finally, the Class I immunogenicity server (http://tools.iedb.org/immunogenicity/, accessed on 18 October 2022) was used to analyze the immunogenicity of these epitopes, and CTL epitopes with immune scores greater than zero were selected. The selected CTL epitopes were identified as immunodominant CTL epitopes for vaccine construction. Finally, sensitization and toxicity of immunodominant CTL epitopes were predicted using the ToxinPred (http://crdd.osdd.net/raghava/toxinpred/, accessed on 18 October 2022), AllerTOP v.2.0 (http://www.ddg-pharmfac.net/AllerTOP/, accessed on 18 October 2022), and Allergen FP v.1.0 (http://ddg-pharmfac.net/AllergenFP/, accessed on 18 October 2022) servers.

### 2.5. Selection of Adjuvants and Guaranty Linkers for Vaccine Construction

Linkers were used to connect the immunodominant CTL, HTL, and B-cell epitopes to construct an MEV against co-infection of MTB and SARS-CoV-2. The KK, AAY, and GPGPG linkers were the most common linkers used in multi-epitope-based vaccine development [1,31]. An adjuvant and pan-HLA DR-binding epitope (PADRE) (amino acid sequence, AGLFQRHGETKATVGEPV) was added to the amino-terminal of the MEV. Adjuvants can enhance the efficacy of vaccines by improving the differentiation of memory cells, effectively inducing the initial immune response [32]. A 50S ribosomal protein L7/L12 (locus RL7 MYCTU) (ID: P9WHE3) was selected as an adjuvant due to its ability to improve the prediction of vaccine immunogenicity. The sequence was obtained from the UniProt database and introduced at the amino-terminal of the MEV via connectors [33].

### 2.6. Toxicity Detection, Solubility Prediction, and Physicochemical Properties of Predicted Vaccine Structures

First, the ToxinPred server, consisting of 1805 hazardous peptides [34], was used to predict the virulence of the vaccine candidates. This server classifies toxic and non-toxic peptides according to a support vector machine model. Then, the Protein-Sol server (https://protein-sol.manchester.ac.uk/, accessed on 20 October 2022) was employed to evaluate the solubility of the vaccine candidates. A prediction score > 0.45 indicated that the solubility of the candidate vaccine was higher than the average experimental solubility in the database, suggesting that the candidate MEV is soluble. Next, the physicochemical properties of the MEV were investigated using the Expasy Protparam server (https://web.expasy.org/protparam/, accessed on 20 October 2022). This server can indicate the theoretical isoelectric point (PI), amino acid composition, molecular weight, aliphatic index, instability, grand average of hydrophilicity (GRAVY), and the full average in vitro and in vivo half-life [35].

### 2.7. Prediction of Immunogenicity, Antigenicity, and Sensitization of Vaccines

The IEDB Immunogenicity server was employed to explore the immunogenicity of the MEV. The antigenicity of the MEV was predicted using the ANTIGENpro (http://scratch.proteomics.ics.uci.edu/, accessed on 25 October 2022) and VaxiJen v2.0 servers. The parameters were set to default values in both servers. Based on cross-validation experiments, the ANTIGENpro server achieves 76% accuracy with the combined dataset [36]. Subsequently, the AllerTOP v.2.0 and Allergen FP v.1.0 servers were used to evaluate the sensitization of the MEV. AllerTOP v.2.0 applies machine learning techniques to classify allergens by exploring the physicochemical properties of proteins. Through five rounds of cross-validation, AllerTOP v.2.0 demonstrated 85.3% accuracy [37]. Finally, AllerFP v.1.0, which has been shown to correctly identify 88% of allergens [38], was used to screen known allergens and non-allergens.

### 2.8. Secondary Structure and 3D Structure Prediction, Optimization, and Verification

The secondary structures of the MEVs were predicted using two servers, the Prabi (https://npsa-prabi.ibcp.fr/cgi-bin/npsa_automat.pl?page=/NPSA/npsa_gor4.html, accessed on 25 October 2022) and PSIPRED (http://bioinf.cs.ucl.ac.uk/psipred/, accessed on 25 October 2022) servers. PSIPRED is a secondary structure generation tool, which can predict folds, transmembrane topology, domain recognition, transmembrane helices, etc. [39]. The Prabi server uses Garnier–Osguthorpe–Robson 4 (GOR4) method to analyze the secondary structure of peptides, and the accuracy of this method has been shown to be about 64.4% [40]. Next, the I-TASSER server (https://zhanggroup.org//I-TASSER/, accessed on 25 October 2022) was used to predict the 3D spatial structure of the vaccine candidates. The 3D spatial structure of the target proteins was simulated using a template structure and comparative similarity index provided by the protein database [41].

Meanwhile, the estimation accuracy of the prediction is based on the modeling confidence score (C-score). Generally, a higher C-score indicates higher accuracy [42]. The GalaxyRefine web server (https://galaxy.seoklab.org, accessed on 25 October 2022) was then used to improve the accuracy of the candidate vaccines [43]. The ProSA web server (https://prosa.services.came.sbg.ac.at/prosa.php, accessed on 25 October 2022) was employed to evaluate the feasibility of the MEV. The frames were entered accurately using the ProSA web server to estimate the exact input structure of the total quality score as a Z-score. When the Z-score exceeded the nature of the native protein, the structure contained errors [44]. Finally, the SWISS-MODEL server (https://swissmodel.expasy.org/assess, accessed on 25 October 2022) was used to draw a Ramachandran diagram for candidate vaccines [45]. The structure evaluation page shows the most relevant scores provided by MolProbity and helps to quickly identify low-quality residues in the structure [44]. The results before and after optimization were compared.

### 2.9. Prediction of Discontinuous B-Cell Epitopes

B-cell epitopes include linear and discontinuous epitopes, but most are discontinuous. Therefore, predicting discontinuous B-cell epitopes is crucial to further refine the spatial structure of the vaccine candidates. The ElliPro server (http://tools.iedb.org/ellipro/, accessed on 26 October 2022), a commonly used nonlinear B-cell epitope prediction server, was selected for prediction, with a screening threshold of 0.693. The epitopes with scores greater than the threshold were established and eventually used as discontinuous B-cell epitopes to construct the MEV [46].

### 2.10. Candidate Vaccine Docked to Toll-like Receptor (TLR) 4 Molecules

TLR is an essential component of innate immunity that recognizes pathogens and initiates the signaling of proinflammatory cytokines. During MTB and SARS-CoV-2 co-infection, host drug resistance depends on TLR4 [47]. Therefore, TLR4 was selected as an adjuvant to the vaccine candidate. The ClusPro 2.0 online server (https://cluspro.bu.edu/home.php, accessed on 26 October 2022) was chosen to perform ligand–receptor docking analysis on candidate vaccines [48] and select the model with the lowest complex binding energy. The palm database (PDB) file of TLR4 used for docking analysis was obtained from the National Center for Biotechnology Information Molecular Modeling Database (PDB ID: 4G8A) (https://www.ncbi.nlm.nih.gov/structure/, accessed on 26 October 2022).

### 2.11. Immune Simulation

The immune simulation was performed using the C-ImmSim server (https://150.146.2.1/C-IMMSIM/index.php, accessed on 1 November 2022), an innovative method for analyzing the immune system [49]. This program provides an immune system simulator with machine-learning techniques for predicting MHC-peptide binding interactions [49]. In this study, all parameters were set to default. In total, 3 injections of 1000 antigens were performed at an interval of 4 weeks, and the level of immune response induced by antigens was measured each time.

### 2.12. Normal Mode Analysis (NMA) of the Complex

NMA of the complex was simulated using the iMODS web server (https://imods.iqfr.csic.es/, accessed on 1 November 2022). The NMA of the complex was verified using the covariance matrix, backbone deformation diagram, eigenvalues, elastic network model, and B-factor values [50].

### 2.13. Codon Optimization

Codon optimization can increase the expression of recombinant proteins, mainly when heterologous expression methods are applied [51]. To express the vaccine candidate, the K12 *Escherichia coli* (*E. coli*) strain was selected as the host organism because it is classified as non-pathogenic to humans [52]. Due to the discrepancy in codon usage between humans and *E. coli*, the GenSmart Codon Optimization (Version Beta 1.0) server (https://www.genscript.com/tools/gensmart-codon-optimization/, accessed on 1 November 2022) was used to improve the codon expression rate by adapting to prokaryotes.

## 3. Results

### 3.1. Selection of Immunodominant Epitopes

HTL epitopes with percentile rank scores < 0.5 were identified using the MHC-II web server. The HTL epitope with the highest ranking according to the antigenicity values > 0.7 and the highest interferon (IFN) prediction score was then selected (Table 1). Then, CTL epitopes with percentile rank scores < 0.5 were identified using the MHC-I web server. The highest-ranked CTL epitopes were then selected according to the immunogenicity score of each antigen. Finally, B-cell epitopes were identified using the ABCpred server to predict the truncation binding score of the epitopes. B-cell epitopes with the scores > 0.85 were selected.

Sensitization and toxicity tests were performed on B-cell, HTL, and CTL epitopes. Finally, the epitopes that passed all tests were selected as candidate immunodominant epitopes, including 10 HTL, 2 CTL, and 4 B-cell epitopes (Table 1).

### 3.2. Primary Structure Construction of the S7D5L4 Vaccine

**Figure 1:**
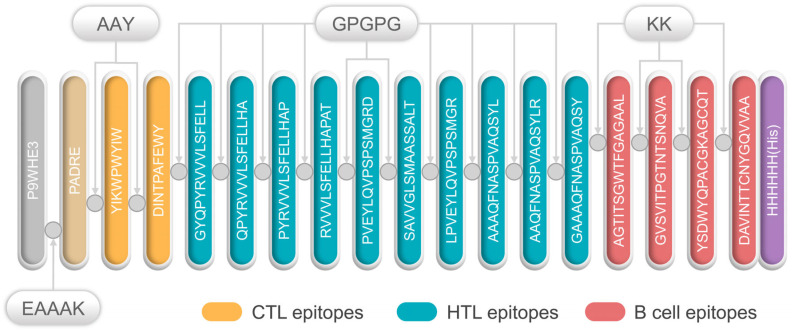
Schematic diagram of the S7D5L4 vaccine. The 454-amino acid peptide sequence contains an adjuvant (light orange) at the amino terminus. It is linked to the pan HLA DR-binding epitope (PADRE) (orange) by the EAAAK linker (indigo blue). The AAY linker is linked to PADRE in a multi-epitope sequence. CTL epitopes (yellow) are ligated using an AAY linker. HTL epitopes (blue) are linked using GPGPG linkers, whereas B-cell epitopes (red) are linked via KK linkers. Finally, a 6x-His tag was inserted into the carboxyl terminus for purification and identification, and the resultant candidate was named, S7D5L4.

All selected epitopes were ligated to construct an MEV (Figure 1). The 50S ribosome (L7/L12) was selected as an adjuvant and ligated with PADRE using an EAAAK linker. Then, the PADRE and CTL epitopes were bound by an AAY linker. At the same time, GPGPG and KK were used to link the HTL epitopes and B-cell epitopes, respectively. Finally, the resultant MEV was named, S7D5L4.

### 3.3. Toxicity Detection, Solubility Prediction, and Physicochemical Properties of the S7D5L4 Vaccine

The physiochemical properties of the S7D5L4 vaccine are summarized in Table 2. Our results show that the S7D5L4 vaccine was non-toxic. In addition, the prediction results of the Protein-Sol server show that the solubility of the S7D5L4 vaccine was 0.462, indicating acceptable solubility. Moreover, the molecular weight of the S7D5L4 vaccine was 46,825.3 Da, with a total of 454 amino acids. Its theoretical PI value was 6.38, suggesting that the vaccine would be acidic. Furthermore, the S7D5L4 vaccine exhibited a half-life of more than 30 h in human reticulocytes (in vitro), more than 20 h in yeast (in vivo), and more than 10 h in *E. coli* (in vivo). In addition, the instability index of the S7D5L4 vaccine was 26.51, indicating that the vaccine was considered stable. Furthermore, its aliphatic index was 80.24 (Table 2), and its GRAVY score was −0.033, demonstrating that the vaccine was hydrophilic (Table 2).

### 3.4. Immunogenicity, Antigenicity, and Sensitization of the S7D5L4 Vaccine

The results show that the predicted antigenicity of the S7D5L4 vaccine was 0.7811 according to VaxiJen v2.0 and 0.4299 according to ANTIENpro. In addition, the immunogenicity of S7D5L4 was 1.045499, according to IEDB Immunogenicity (Table 2). Therefore, the S7D5L4 vaccine had acceptable immunogenicity and antigenicity. At the same time, an ideal vaccine must be non-allergenic. Therefore, the AllerTOP v.1.0 and AllergenFP v.2.0 servers were used to predict the allergenicity of the S7D5L4 vaccine, and the results show that the S7D5L4 vaccine was non-allergenic (Table 2).

### 3.5. Secondary Structure Prediction and 3D Structure Modeling of the S7D5L4 Vaccine

**Figure 2:**
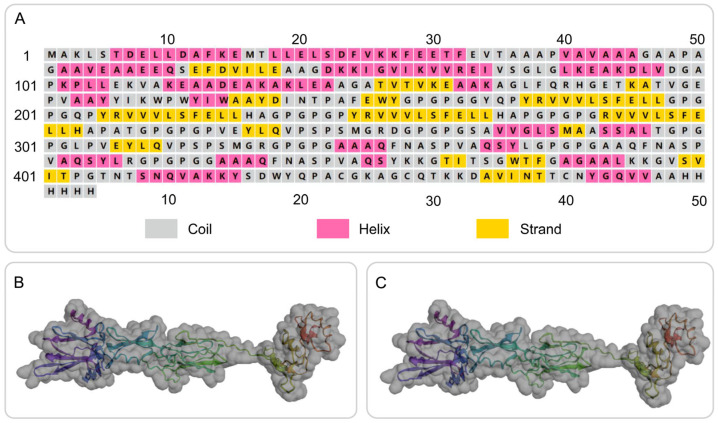
Secondary and tertiary structure prediction of the S7D5L4 vaccine. (**A**) I-TASSER predicted 3D models of vaccine candidates. (**B**) Optimized 3D model using I-TASSER. (**C**) The selected vaccine contained 31.72% α-helixes, 20.70% β-strands, and 47.58% random coils. The amino acid sequence of the S7D5L4 vaccine was indicated in purple to red from the N-terminal to the C-terminal.

The secondary structure of the S7D5L4 vaccine was successfully simulated using the PSIPRED and Prabi servers (Figure 2A). The S7D5L4 vaccine included 31.72% α-helixes, 20.70% β-strands, and 47.58% random coils. Subsequently, the 3D structural model of the S7D5L4 vaccine was designed using the I-TASSER server, and five 3D models were successfully predicted, with Z-scores ranging from 1.08 to 4.52. The C-score of each model was −1.9, −2.35, −2.99, −3.08, and −3.21, with higher C-scores indicating higher confidence [41]. Therefore, the best model with a C-score of −1.9 was selected to simulate the 3D structure of S7D5L4 (Figure 2B). The template modeling score (TM-score) of the chosen model was 0.49 ± 0.15, and the root-mean-square deviation (RMSD) was 11.6 ± 4.5. The TM-score was developed as a scale to compare similarities between structures [53] and is generally recommended for solving RMSD problems by circumventing the sensitivity of RMSD to local inaccuracies.

### 3.6. Optimization of the Tertiary Structure of the S7D5L4 Vaccine

**Figure 3:**
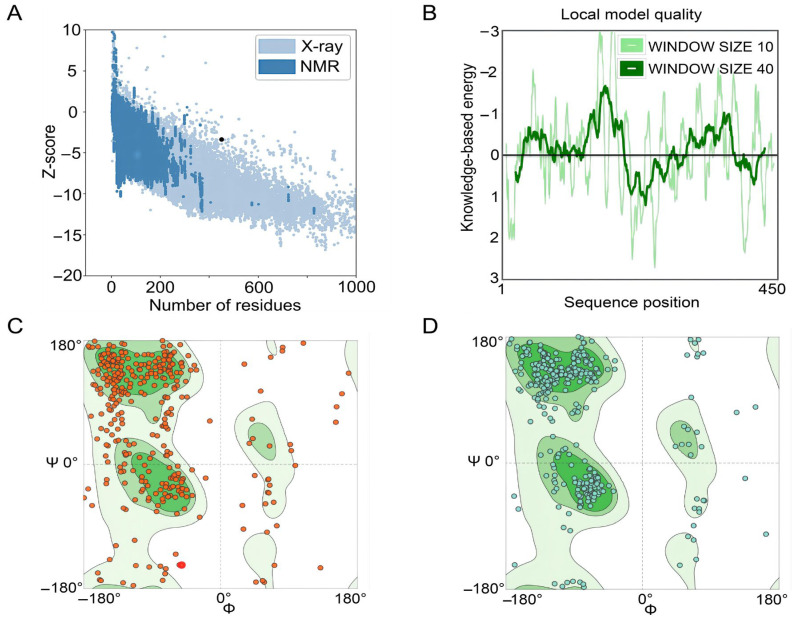
Evaluation and validation of tertiary structure models of the S7D5L4 vaccine. (**A**) The ProSA web server provided a Z-score of −3.36. (**B**) Tertiary structure energy map of S7D5L4 validated by the ERRAT web server. (**C**) The Ramachandran plot shows the following composition: favored region, 65.62%; outlier region, 13.84%; and rotamer region, 14.85%. The favored, outlier, and rotamer regions were shown in dark green, green, and light green, respectively. Amino acid residues before optimization were indicated by red dots. (**D**) The optimized Ramachandran plot shows the following composition: favored region, 81.03%; outlier region, 4.24%; and rotamer region, 0.61%. The favored, outlier, and rotamer regions were shown in dark green, green, and light green, respectively. Amino acid residues after optimization were indicated by blue dots.

The GalaxyRefine web server was used to refine the 3D model of the S7D5L4 vaccine. The server can achieve higher-quality prediction results by improving the consistency of the structure. Through energy minimization and loop refinement, five optimized 3D models were obtained again. Model 3 was selected for further study (Figure 2C), as it was considered optimal. The GDT-HA (sequence-dependent superposition scores with the high accuracy version), RMSD, MolProbity, Clash score, low rotamers, and Rama-favored values of model 3 were 0.9094, 0.545, 2.629, 26.3, 0.6, and 81.0, respectively. Next, the ERRAT and ProSA web servers were used to verify the errors and quality of the model. The results show that the optimized Z-score was −3.36 (Figure 3A). After optimization, the overall quality factor of the model was improved to 64.3392%. The energy diagram is shown in Figure 3B. The Ramachandran plot score shows that the rotamer region was 14.85%, the outlier region was 13.84%, and the favored region was 65.62% before optimizing the vaccine candidates (Figure 3C). After optimization, the rotamer region changed to 0.61%, the outlier region was 4.24%, and the favored region was 81.03% (Figure 3D). Therefore, the favored region of the S7D5L4 vaccine was significantly improved from 65.62 to 81.03%.

### 3.7. Discontinuous B-Cell Epitopes

**Figure 4:**
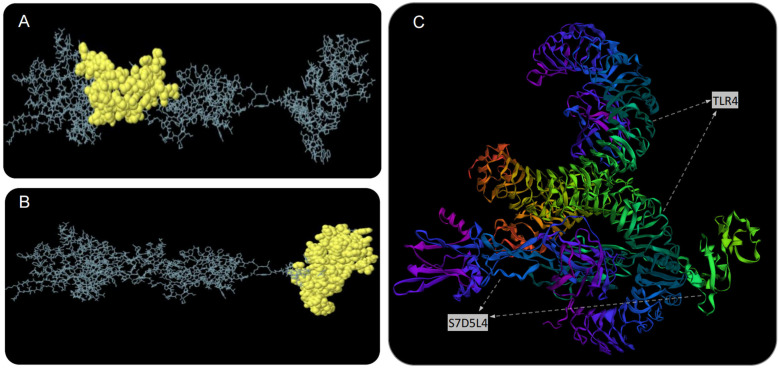
Discontinuous B-cell epitope prediction and molecular docking simulation of the S7D5L4 vaccine. Yellow figures in (**A**,**B**) represent two discontinuous B-cell epitopes. The rest of the amino acid sequences were shown in gray. (**C**) The model with the lowest complex binding energy was selected for demonstration and subsequent analysis, and the docking results of S7D5L4 and TLR4 were simulated using ClusPro 2.0.

The S7D5L4 vaccine was analyzed using the ElliPro server for discontinuous B-cell epitopes. The results show that three discontinuous B-cell epitopes were successfully predicted. For predicted discontinuous B-cell epitopes, a prediction model with a score >0.69 is usually selected. Therefore, we finally selected two discontinuous B-cell epitopes with scores of 0.693 (Figure 4A) and 0.729 (Figure 4B). The amino acid fragments of the selected epitopes are shown in Table 3.

### 3.8. The S7D5L4 Vaccine Docked to TLR4 Molecule

We used the ClusPro 2.0 server to doc the S7D5L4 vaccine with TLR4 and generated 30 predicted complexes. According to the prediction results, the top 10 models with the lowest complex binding energies were extracted (Table 4). Finally, the model with the weakest complex binding energy (Cluster: 0) (Figure 4C) was selected for subsequent analysis and optimization.

### 3.9. Immune Simulation of the S7D5L4 Vaccine

**Figure 5:**
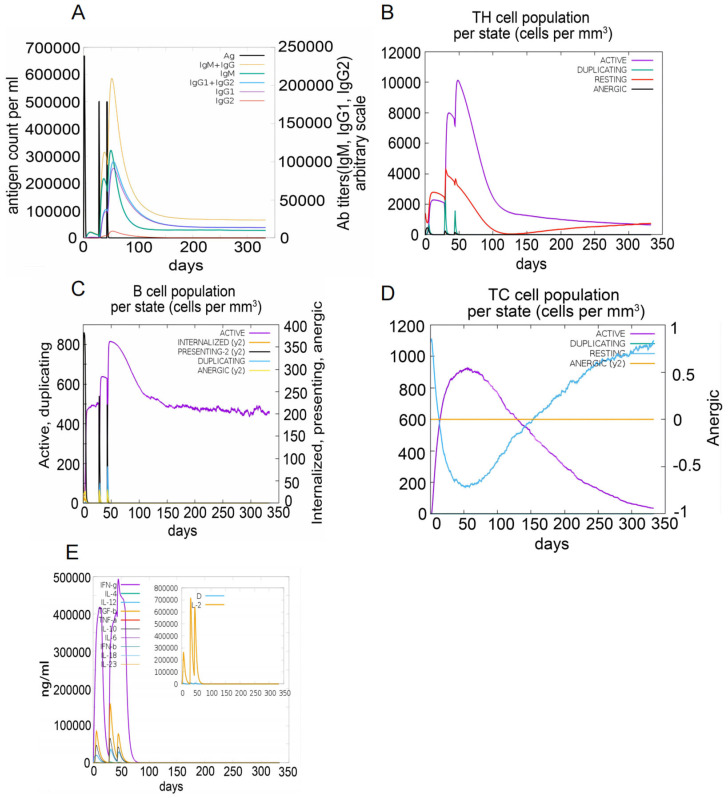
The immune responses induced by the S7D5L4 vaccine in the C-ImmSim server. (**A**) The primary B-cell antibodies produced after antigen stimulation, IgM + IgG (yellow), over time. (**B**) Changes in the secretion levels of HTLs. (**C**) Secretion by active B cells (purple) after antigen stimulation. (**D**) Changes in the secretion levels of CTLs. (**E**) Change in cytokine secretion levels, mainly IFN-γ (purple) and IL-2 (yellow).

The C-ImmSim server was used to simulate immune responses. The S7D5L4 vaccine successfully stimulated the innate immune system and induced immune responses in the simulation. The results show that the S7D5L4 vaccine could induce B cells to produce high levels of IgM and IgG antibodies (Figure 5A), suggesting the establishment of immune memory. Similarly, high responses with corresponding memory development were observed in T-helper and cytotoxic T-cell populations (Figure 5B,D). We found that the population of active cytotoxic T lymphocytes gradually increased and began to decline after reaching a peak on day 60 after stimulation. However, resting cytotoxic T lymphocytes showed the opposite trend (Figure 5D). We also observed that the S7D5L4 vaccine promoted substantial proliferation of active B lymphocytes (Figure 5C). Moreover, the S7D5L4 vaccine induced high levels of IFN-γ and IL-2 with repeated exposure injections at four-week intervals (Figure 5E).

### 3.10. NMA of the Complex

**Figure 6:**
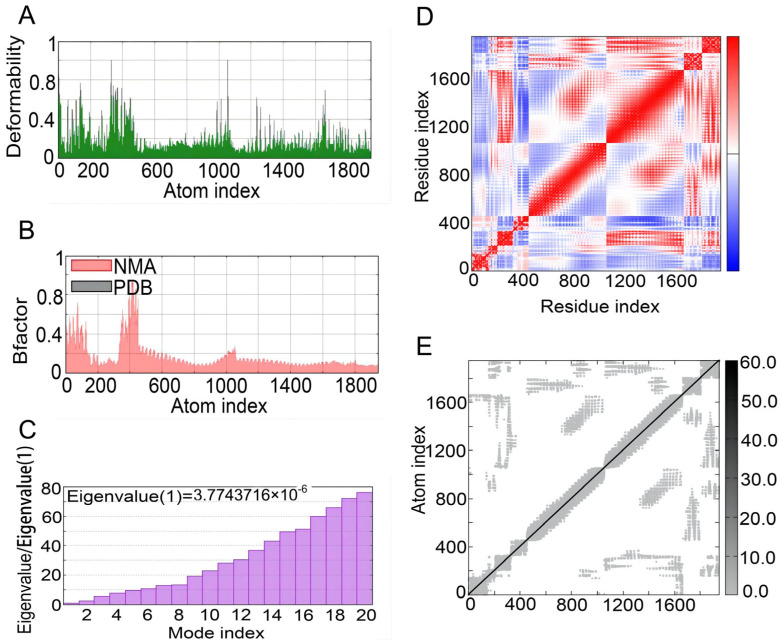
NMA modeling of S7D5L4-TLR4 complexes. (**A**) Modeling the deformability of the main chain. (**B**) B-factor values were calculated using NMA to quantify the uncertainty of all atoms. (**C**) The eigenvalues represent the energy required to deform the structure between adjacent complexes. (**D**) Covariance matrix between residues (red: correlated, blue: anti-correlated, white: uncorrelated). (**E**) Elastic network model between springs and atoms. Darker gray indicates stiffer spring.

The iMOD server was used to perform NMA to evaluate the physical mobility and stability of the docking complex between the S7D5L4 vaccine and TLR4 receptor [54]. The hinge position was visualized using high strain (Figure 6A). The ratio between the RMSD and B factor values was calculated using NMA (Figure 6B). In addition, the eigenvalue of the complex was 3.7743716 × 10^−6^, and the energy dissipation during structural deformation was closely related to the eigenvalue (Figure 6C). Furthermore, the correlation between residue pairs was detected in the covariance matrix (Figure 6D). Finally, the elastic network model assumed connections between atoms and springs, confirming the stability of the S7D5L4 model (Figure 6E).

### 3.11. Codon Optimization

The GenSmart Codon Optimization (Version Beta 1.0) server was used to optimize the codon of S7D5L4 in *E. coli*. The average guanine and cytosine content of the optimized codon was 59.84%, indicating that the S7D5L4 vaccine might be expressed in an *E. coli* host, as the ideal guanine and cytosine content ranged from 30 to 70%.

## 4. Discussion

The spread of SARS-CoV-2 through droplets has seriously threatened world health security [55]. Moreover, the co-infection of SARS-CoV-2 with MTB is a proven risk factor for increased mortality and severity, and the specific mechanism remains unclear [56]. Immunoinformatic predictions expand the breadth of the vaccine design environment, which may provide a solution to prevent MTB co-infection with SARS-CoV-2. The current revolution in sequencing technology and the establishment of genomes for various pathogens lead to the continuous development of methods for designing vaccines. Furthermore, immunoinformatics for vaccine development will save time and money on research [57].

Meanwhile, the development of cloud servers and computing tools to process proteomics and genomics has further changed the research status of computerized vaccine prediction to include fungi, bacteria, viruses, and even cancer [58]. Therefore, the use of immunoinformatics for epitope vaccine prediction is preferrable. In this study, we obtained the amino acid sequences of three candidate antigens from the UniProt server and subsequently carried out model building, optimization, and evaluation. During the vaccine design phase, we added necessary TLR adjuvants, improving the stability, immune response, and longevity of the S7D5L4 vaccine [59]. The addition of TLR adjuvants also enables S7D5L4 to recognize cognate TLR ligands, even those that come from different immune cells, and induce immune responses by stimulating naive CD4^+^ T cells [60]. Furthermore, we used GPGPG, AAY, and KK linkers to successfully connect epitopes. Linkers are indispensable structures in vaccine construction and can aid in vaccine expression, folding, and stability [61]. For example, the AAY linker influences the stability of participating structures by providing proteasome cleavage sites [62]. In addition, KK linkers can help maintain the independent immunogenic activity of epitopes [63]. Finally, we obtained the complete sequence and structure of the S7D5L4 vaccine.

The molecular weight of S7D5L4 was 46,555.01 Da, and the predicted solubility value was 0.462, indicating that the S7D5L4 vaccine had acceptable quality and solubility [64]. In addition, the instability index of the S7D5L4 vaccine was 26.51, which is far less than the threshold value of 40. Previous studies have shown that proteins with an instability index greater than 40 must be unstable [65]. Additionally, the aliphatic index of the S7D5L4 vaccine was 80.24, indicating that the side chains were composed of aliphatic groups, and the S7D5L4 vaccine had high thermal stability potential. For proteins with molecular weights less than 100,000 Da, the aliphatic index is an important index to evaluate the thermal stability potential of the protein [66]. Interestingly, we found that the S7D5L4 vaccine was characterized by non-toxicity, non-sensitization, good antigenicity, and immunogenicity. Overall, these characteristics of the S7D5L4 vaccine ensure that it could be a worthy candidate for subsequent in vivo validation.

The MEV prediction strategy was adopted in this experiment, which has more advantages than the whole-protein strategy for vaccine design. MEVs can stimulate an immune response to epitopes but do not induce systemic autogenous immune responses [67]. In this study, T- and B-cell epitopes were included, enabling cellular and humoral responses. As we did not include the whole protein, the associated side effects of the non-essential epitopes produced by the entire protein antigen were minimized. This method may also stimulate the generation of protective and specific immune responses to the greatest extent [68], which was confirmed in our immune stimulation, in which the S7D5L4 vaccine induced cellular and humoral immunity. In this study, the IFN-γ release score of HTL epitopes was also predicted, and the epitope with positive IFN-γ release and the highest score was finally selected. Adequate IFN-γ release from CD4^+^ and CD8^+^ T cells is crucial for controlling concurrent MTB infections [69]. A drawback of most current neutralizing antibodies is that those without T-cell immunity cannot provide lasting immunity and protection. Therefore, T-cell immunity is essential [70]. For SARS-CoV-2, the specific memory responses of the CD4^+^ and CD8^+^ T cells were more persistent. In conclusion, the S7D5L4 vaccine showed good biological properties and structure and should undergo subsequent in vitro and in vivo verification. This vaccine should be continuously optimized until it is successfully used to prevent the co-infection of MTB and SARS-CoV-2.

Nevertheless, this study had several limitations. First, the S7D5L4 vaccine consisted of TLR4 but not TLR2, which might decrease its ability to activate the MyD88-NK-κB signaling pathway. Second, the physicochemical and immunological characteristics of the S7D5L4 vaccine were only investigated in silico; thus, verification through in vitro and in vivo experiments is lacking.

## 5. Conclusions

In this study, we designed a multi-epitope vaccine, S7D5L4, using an immunoinformatic online server, among other tools. The S7D5L4 vaccine comprised 10 HTL, 2 CTL, and 4 B-cell epitopes, in addition to adjuvants. The results show that the S7D5L4 vaccine was characterized by high antigenicity, immunogenicity, non-sensitization, non-toxicity, good solubility, and stability. In addition, the S7D5L4 vaccine had good affinity for TLR4 and induced a high level of innate and adaptive immune responses in silico.

## Figures and Tables

**Table 1:** Detailed information of the HTL, CTL, and B-cell epitopes used to construct the S7D5L4 vaccine.

Protein	Peptide Sequence	Length	Alleles	Percentile Rank ^a^	Antigenicity Score ^b^	IFN-γ Score ^c^	Immunogenicity Score ^d^	ABC Pred Score ^e^	AllerTOP V 1.0	AllergenFP v.2.0	Toxin Pred
HTL epitopes											
S protein											
	GYQPYRVVVLSFELL	15	HLA-DPA1*01:03/DPB1*02:01	0.36	1.074	0.6533982			Non ^f^	Non ^f^	Non ^f^
	QPYRVVVLSFELLHA	15	HLA-DPA1*01:03/DPB1*02:01	0.36	0.9109	0.60855322			Non	Non	Non
	PYRVVVLSFELLHAP	15	HLA-DPA1*03:01/DPB1*04:02	0.25	0.8161	0.56872818			Non	Non	Non
	RVVVLSFELLHAPAT	15	HLA-DRB1*01:01	0.24	0.7485	0.5092653			Non	Non	Non
Ag85a											
	PVEYLQVPSPSMGRD	15	HLA-DRB1*04:01	0.46	0.7094	0.99952242			Non	Non	Non
	SAVVGLSMAASSALT	15	HLA-DRB1*09:01	0.34	0.6602	0.86704792			Non	Non	Non
	LPVEYLQVPSPSMGR	15	HLA-DRB1*04:01	0.38	0.7922	0.82782141			Non	Non	Non
Mtb8.4											
	AAAQFNASPVAQSYL	15	HLA-DRB1*09:01	0.34	0.6839	0.45466067			Non	Non	Non
	AAQFNASPVAQSYLR	15	HLA-DRB1*09:01	0.34	0.6176	0.56966616			Non	Non	Non
	GAAAQFNASPVAQSY	15	HLA-DRB1*09:01	0.35	0.5933	0.39892204			Non	Non	Non
CTL epitopes											
S protein											
	YIKWPWYIW	9	HLA-A*23:01	0.1	0.9673		0.42524		Non	Non	Non
Ag85a											
	DINTPAFEWY	10	HLA-A*26:01	0.24	1.8593		0.38838		Non	Non	Non
B-cell epitopes											
S protein											
	AGTITSGWTFGAGAAL	16						0.97	Non	Non	Non
	GVSVITPGTNTSNQVA	16						0.95	Non	Non	Non
Ag85a											
	YSDWYQPACGKAGCQT	16						0.93	Non	Non	Non
Mtb8.4											
	DAVINTTCNYGQVVAA	16						0.86	Non	Non	Non

^a^ The percentile ranking of the selected epitopes: epitopes with ranking score <0.5 were selected. ^b^ The antigenicity score: epitopes with antigenicity score >0.5 were selected. ^c^ IFN-γ score: epitopes with a positive score and the highest score were selected. ^d^ The immunogenicity score: epitopes were selected in order of score. ^e^ The linear B-cell epitope prediction score: epitopes were selected in order of score. ^f^ Non: no sensitization or toxicity.

**Table 2:** Prediction of S7D5L4 parameters.

Parameters		Results
Biological characteristics		
	Antigenicity	0.7811 ^a^
		0.4299 ^b^
	Immunogenicity	1.45499
Half-life (h) ^c^		
	Mammalian reticulocytes (in vitro)	>30
	Yeast (in vivo)	>20
	*E. coli* (in vivo)	>10
Physicochemical properties		
	Molecular weight (Da)	46,825.3
	Number of amino acids	454
	Isoelectric point	6.38
	Instability index	26.51
	Fat index	80.24
Basic features		
	Toxicity	Non-toxic
	Sensitization	Non-allergenic
	Solubility	0.462
	GRAVY score	−0.033

^a^ The score of antigenicity predicted using the VaxiJen v2.0 server. ^b^ The score of antigenicity predicted using the ANTIENpro server. ^c^ The half-life of a candidate sequence in hours.

**Table 3:** The discontinuous B cell epitopes residues of the S7D5L4 predicted by the ElliPro.

No	Residues	Number of Residues	Score
1	A:G331, A:P332, A:G333, A:A341, A:A345, A:Q346, A:S347, A:A349, A:A350, A:Y351, A:Y352, A:I353, A:K354, A:W355, A:P356, A:W357, A:Y358, A:I359, A:W360, A:A361, A:A362, A:Y363, A:I365, A:N366, A:T367, A:P368, A:A369, A:F370, A:E371, A:Y373, A:K374, A:K375, A:G376, A:T377, A:I378, A:T379, A:S380, A:G381, A:W382, A:T383, A:F384, A:G385, A:A386, A:G387, A:A388, A:A389, A:L390, A:K391, A:G393, A:V394, A:S395, A:V396, A:I397, A:T398, A:P399, A:G400, A:T401, A:N402, A:T403, A:S404, A:N405, A:Q406, A:V407, A:A408, A:K409, A:K410, A:Y411, A:S412, A:D413, A:Y415, A:Q416, A:P417, A:A418, A:C419, A:G420, A:K421, A:A422, A:G423, A:C424, A:Q425, A:T426, A:K427, A:K428, A:D429, A:A430, A:V431, A:I432, A:N433, A:T434, A:T435, A:C436, A:N437, A:Y438, A:G439, A:Q440, A:V441, A:V442, A:A443, A:A444, A:H445, A:H446, A:H447, A:H448, A:H449, A:H450	105	0.792 ^#^
2	A:Q135, A:H136, A:H137, A:G138, A:E139, A:G140, A:A143, A:T144, A:V145, A:G146, A:E147, A:P148, A:V149, A:E150, A:K151, A:Y158, A:V160, A:V161, A:V162, A:L163, A:G169, A:P170, A:G171, A:P172, A:G173, A:Q174, A:P175, A:Y176, A:R177, A:V178, A:V179, A:V180, A:L181, A:S182, A:F183, A:E184, A:L185, A:L186, A:H187, A:A188, A:G189, A:P190, A:G191, A:P192, A:G193, A:P194, A:Y195, A:R196, A:V197, A:V198, A:V199, A:L200, A:S201, A:F202, A:E203, A:L204, A:L205	84	0.693 ^#^

^#^ Only epitopes with a score greater than 0.69 could be accepted, so No 1 and No 2 were selected.

**Table 4:** Prediction results of docking of S7D5L4 with TLR4 molecules ^a^.

Cluster	Members	Representative	Weighted Score
0	27	Center	**−921.5** **^b^**
		Lowest Energy	**−1208.1** **^b^**
1	27	Center	−1004
		Lowest Energy	−1072.3
3	20	Center	−962.3
		Lowest Energy	−1058.4
4	16	Center	−1057.3
		Lowest Energy	−1057.3
11	13	Center	−883.5
		Lowest Energy	−1031.6
13	12	Center	−932.2
		Lowest Energy	−1114.5
14	12	Center	−1088.4
		Lowest Energy	−1143.5
15	12	Center	−995.9
		Lowest Energy	−1040.1
22	10	Center	−1014.6
		Lowest Energy	−1014.6
29	9	Center	−1089
		Lowest Energy	−1089

^a^ Thirty models were successfully predicted, and the top 10 models are shown according to the lowest complex binding energy. ^b^ The model ranked lowest in terms of complex binding energy was therefore selected for subsequent analysis (bold font).

## Data Availability

All data generated or analyzed during this study are included in this published article.

## Abbreviations

TB: tuberculosis; SARS-CoV-2: severe acute respiratory syndrome coronavirus-2; MTB: *Mycobacterium tuberculosis*; HTL: helper T lymphocyte; CTL: cytotoxic T lymphocyte; TLR: toll-like receptor; MHC: major histocompatibility complex; MEV: multi-epitope vaccine; IEDB: immune epitope database; *E. coli*: *Escherichia coli*; HLA: human leukocyte antigen; IFN: interferon; PADRE: pan HLA DR-binding epitope; NMA: normal mode analysis; GRAVY: grand average of hydrophilicity; PDB: palm database; PI: isoelectric point; RMSD: root-mean-square deviation; IgM: immunoglobulin-M; IgG: immunoglobulin-G; TH: T helper; TC: T cytotoxic; IL-2: interleukin-2.
